# Beneficial Effects of Daily Consumption of Garlic and Onion Extract Concentrate on Infectious Respiratory Diseases in Elderly Resident Volunteers

**DOI:** 10.3390/nu15102308

**Published:** 2023-05-15

**Authors:** Jorge García-García, Carlos Gracián, Alberto Baños, Enrique Guillamón, Julio Gálvez, Alba Rodriguez-Nogales, Juristo Fonollá

**Affiliations:** 1 Department of Pharmacology, Center for Biomedical Research (CIBM), University of Granada, 18071 Granada, Spain; jgarcia.51@ugr.es (J.G.-G.); albarn@ugr.es (A.R.-N.); 2 Nursing Home “Residencia de Mayores Claret”, 18011 Granada, Spain; cgracianalcaide6@gmail.com; 3 DMC Research Center, 18620 Granada, Spain; abarjona@dmcrc.com (A.B.); enrique.guillamon@dmcrc.com (E.G.); juristo@ugr.es (J.F.); 4 Instituto de Investigación Biosanitaria de Granada (ibs.Granada), 18012 Granada, Spain; 5 Centro de Investigación Biomédica en Red–Enfermedades Hepáticas y Digestivas (CIBER-EHD), 28029 Madrid, Spain

**Keywords:** onion, garlic, organosulfur compounds, dietary supplement, immunostimulant, elderly

## Abstract

Aging is a biological process with high susceptibility to several infections. This risk increases in older patients in residential care facilities (RCF). Thus, there is a clear demand for developing preventive interventions with new therapeutic compounds that combine efficacy and safety. This could be the case of compounds derived from plants of the genus *Allium* spp. The purpose of this study was to evaluate the impact of a combination of a garlic and onion extract concentrate standardized in organosulfur compounds derived from propiin on the incidence of respiratory tract infections in elderly patients of RCF. Sixty-five volunteers were selected at random to receive a placebo or a single daily dose of the extract for thirty-six weeks. Different clinical visits were performed to evaluate the main respiratory diseases with an infectious origin, as well as the associated symptoms and their duration. The extract showed a clinical safety profile and significantly reduced the incidence of respiratory infections. Moreover, the treatment decreased the number and duration of the associated symptoms compared with the placebo group. For the first time, we demonstrated the protective effect of *Alliaceae* extract in respiratory infectious diseases in elderly healthy volunteers, which could be used prophylactically against the most common infectious respiratory diseases.

## 1. Introduction

Aging is a natural process that impairs several physiological systems, including the immune system, resulting in increased susceptibility to infectious, auto-immune, and neoplastic diseases. The world population over the age of 65 is expected to reach 1.5 billion in 2050 [1]. Therefore, emphasis on the overall quality of life of the older person represents a major global public health challenge. Most older adults would prefer to age at home; however, unfortunately, this goal is frequently complicated due to the loss of cognitive and physical abilities, autonomy, and independence. Furthermore, the traditional family structure and socialization have changed, and consequently, home-based care for older adults is declining, and the demand for residential care facilities (RCFs) is rapidly increasing. 

RCFs have become receptors of patients with a high risk of acquiring infections, which are mainly related to their underlying conditions and the invasive procedures they may undergo. In fact, it is well known that older adults who reside in RCFs are highly vulnerable to respiratory tract infections [2,3,4]. Among them, pneumonia, influenza, and recently, severe acute respiratory syndrome coronavirus 2 (SARS-CoV-2) are common and can endanger their life. Although vaccination against these pathogens in this population is widespread, outbreaks persist, resulting in continued morbidity and mortality. Moreover, it has been reported that once infected, the residents are more likely to develop complications, such as lower respiratory tract illness, which is the most important cause of death in elderly residents, and one of the most common reasons for transfer to the hospital [5]. Therefore, there is clearly an urgent need to develop potential and preventive therapeutic strategies that reduce the incidence and/or the severity of respiratory infections. In this context, different plant-based natural herbal formulations and related compounds, which have been traditionally used for the protection against infections and for the improvement of the immune response, are being assayed for the discovery and development of novel antiviral drugs. Since most of these plant-derived products have been reported to be safe after their administration, they could become a high-value therapeutic approach for these conditions in this vulnerable population [6].

In this context, plants of the genus *Allium* spp. such as garlic (*Allium sativum* L.) and onion (*Allium cepa* L.), which are rich in organosulfur compounds, including allicin, propyl-propane thiosulfinate (PTS), and propyl-propane thiosulfonate (PTSO), have been traditionally used against conditions such as joint inflammation, constipation, infectious diseases, and parasitic infestations [7,8,9]. Most of these beneficial effects have been confirmed experimentally in vivo and in vitro [10]. Additionally, in vitro studies have reported that the supplementation of a formula that includes concentrated garlic and onion shows broad-spectrum antimicrobial activity against multidrug-resistant bacteria isolated from human samples [11], and is also effective against porcine reproductive and respiratory syndrome virus (PRRS), as reported in experimental and human studies [12].

Therefore, the aim of this study was to evaluate the impact of daily consumption of a combination of garlic and onion extract concentrate rich in organosulfur compounds, severity, and duration of respiratory tract infections in healthy elderly volunteers who live in a nursing home.

## 2. Materials and Methods

### 2.1. Ethics, Approval, and Consent

This study was performed in compliance with the Declaration of Helsinki Ethical Principles for Medical Research involving human subjects and its amendment, and the Guidelines on Good Clinical Practice standards of CPMP/ICH/135/95 and ISO 14155 and all relevant Spanish guidelines. The study protocol was reviewed and approved by the Regional Ethical Committee (Granada, Spain) and registered in the US Library of Medicine (http://www.clinicaltrials.gov, (accessed on 1 February 2022)) with the ID NCT04647071.

Before the start of the study, the medical team of the residence selected the participants due to their characteristics, and they were invited to participate in the study. If they agreed, their relatives were informed so that they could also give their consent. All subjects provided written informed consent prior to their inclusion in the study.

### 2.2. Subjects and Study Design

The study enrolled 65 healthy older volunteers of both sexes who lived in the nursing home Residencia de Mayores Claret in Granada (Spain), aged more than 65 years and were vaccinated against flu according to the inclusion criteria. Exclusion criteria were, before the study, having any disease that affects the development and results of the study, being unable to understand the study and sign voluntarily and freely the informed consent, and having a low expectation of compliance with the study protocol; during the study, and always according to the opinion of the researchers, failure to comply with the study protocol, suffering some adverse event not tolerated by the subject, suffering changes in health status incompatible with continued participation in the study, and wish to withdraw from the study voluntarily. Finally, during the development of the study, the residence offered all volunteers the opportunity to be vaccinated against the SARS-CoV-2 virus. Agreeing to be vaccinated was a mandatory condition to continue in the study.

**Figure 1:**
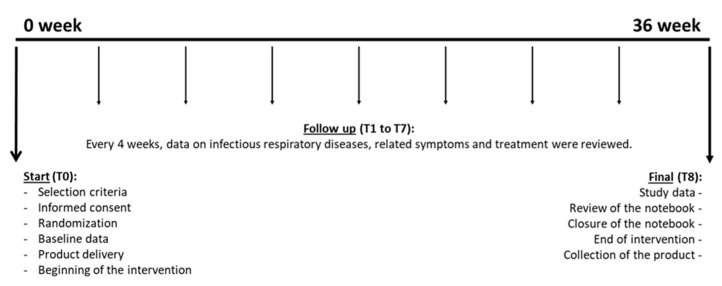
Representation of the experimental design of the study from week 0 until week 36. Procedures carried out at each time (T) are shown.

A controlled, randomized, double-blind, 2 parallel-group study was carried out for 36 weeks during the months with the highest incidence of respiratory diseases (October 2020–June 2021). Eligible volunteers were equally assigned to the product or the placebo group (33 subjects in the active group and 33 in the placebo group), according to a previously prepared randomization list generated using free software www.randomization.com. Accordingly, clinical visits were performed at the beginning of the study (T0) and every four weeks (T1-T7) until the end of the study (T8) (Figure 1).

### 2.3. Intervention

Participants were randomized to receive either the active or a placebo product. The active product (Aliocare^®^, DOMCA SAU., Granada, Spain) contained concentrated onion extract (86 mg) standardized in organosulfur compounds derived from propiin (10 mg per capsule), garlic powder (14 mg), and microcrystalline cellulose (9892-Capsucel^®^, Laboratorios Guinama, La Pobla de Vallbona, Valencia, Spain) up to 450 mg, whereas the placebo product only contained 450 mg of microcrystalline cellulose. Both were delivered in hydroxypropyl methylcellulose capsules (Solchem^®^, Solchem, Barcelona, Spain). The medical team at the nursing home was in charge of administering one capsule a day to each volunteer during lunch for the 36 weeks, ensuring that they ingested it.

Participating subjects were instructed not to deviate from their regular habits during the intervention. Moreover, the participants’ diet was controlled throughout the study (the volunteers had to maintain their menu type).

Neither the researchers (intervention and statistical analysis) nor the participants knew which treatment sequence the subjects had been assigned to; the researchers were unblinded only at the end of the study. The odor of the product was masked to ensure blindness.

### 2.4. Clinical Parameters

The study’s primary outcome was the incidence of respiratory diseases of infectious origin (RDIO), mainly influenza-like illness (ILI), the common cold, and COVID-19. The diagnosis of ILI was made following the guidelines of the European Center for Disease Prevention and Control [13] (Influenza case definitions (2017)) as follows: sudden onset of symptoms with one or more respiratory symptoms (cough, sore throat and/or nasal congestion) plus one or more systemic symptoms (fever, headache, myalgia and/or malaise). The common cold was determined by the sudden onset of one or more respiratory symptoms (cough, sore throat, and/or nasal congestion) without systemic symptoms [14]. Since many of these symptoms coincide with those of infection by the SARS-CoV-2 virus, all those suspected of infectious respiratory disease underwent a PCR (polymerase chain reaction) test. In the case of a negative result, ILI or a common cold was diagnosed.

For this reason, every 28 days during the study, 14 clinical parameters related to these illnesses were monitored. The chosen symptoms were cough, fever, nasal congestion, throat pain, headache, bone pain, fatigue, chest pain, difficulty breathing, loss of smell/taste, nausea, diarrhea, lack of appetite, and sleeping problems. Moreover, medication intake and total illnesses were followed during the intervention. The events were recorded daily by the medical team in the residence files and collected every four weeks in the study database.

The severity of the events was analyzed by studying the number of symptoms experienced by the volunteers and their duration.

Finally, product safety was monitored daily by asking volunteers if they felt bad when taking the product and by weighing and measuring blood pressure every 28 days. Medicine intake was also followed.

### 2.5. Statistical Analysis

The calculation of the sample size, as well as the statistical analysis, was carried out by the company SEPLIN Soluciones Estadisticas, S.L. (Granada, Spain). The blind of the study was only opened by the principal investigator upon receipt of the statistical report.

A study was proposed with a sample size necessary to determine a difference in the reduction of incidences of respiratory symptoms of 35% and determining a statistical power of 80% and a significance level of 5%. Based on these considerations, 31 patients per group would be necessary, making a total of 62. In any case, in anticipation of possible losses during the study, 69 volunteers were recruited.

The results of the primary variable “Incidence of respiratory symptoms associated with infections” were analyzed longitudinally at different times (every four weeks of the study) and according to the treatment groups. Descriptive measures of the incidences (IRR Incidence rate ratio/OR-odds ratio), 95% confidence interval for the IRR/OR by study group, and time were studied, depending on the number of respiratory events or the occurrence of at least one case of respiratory symptom.

Generalized Mixed Models or repeated measures were used to compare the groups over time, adjusted for the baseline variables and the characteristics of the person both for the occurrence of some symptom at each measurement moment and the response of the number of events that occurred (longitudinal analysis of the response; mixed logistic model in the case of occurrence and mixed Poisson model in the case of the number of observed events).

To analyze the duration of the symptoms (number of days with symptoms associated with each event), these quantities have been described considering means, median, deviation, and range of values. The results have been compared between groups at each moment as well as the global measurement of total days of symptoms at the end of the study. A Poisson model has been applied to analyze the number of days with symptoms over time, comparing the mean number of days with symptoms by group.

## 3. Results

### 3.1. Study Data, Compliance, and Baseline Characteristics of the Subjects

**Figure 2:**
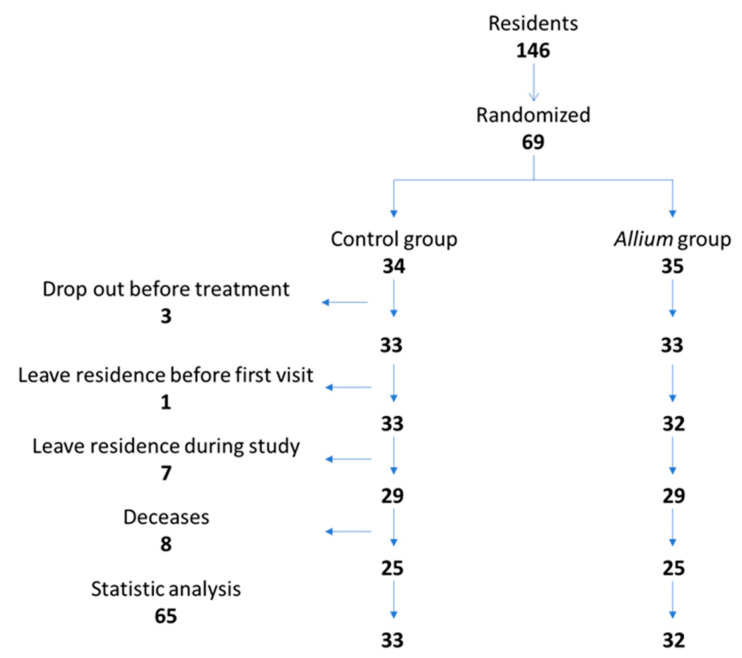
Flow chart of the subjects included in the study.

During the period from October 2020 to June 2021, 66 healthy individuals were enrolled and randomly distributed into 2 groups: the treated group that received garlic and onion concentrated extract (33 subjects) and the control group that received the placebo (33 subjects) (Figure 2). The product was administered under medical supervision during lunch, achieving 100% compliance.

The demographic and clinical characteristics of the subjects at the beginning of the study were similar in both study groups (Table 1), and no significant differences were observed between the two groups for any of the analyzed variables. Most participants were older than 80 years, with a mean value of 84.4 ± 9.0 years, and the majority were female (80%). It is noteworthy that there was a high percentage of smokers (86.2%) and regular drinkers (92.3%). Despite this, blood pressure values were within normal limits due to the habitual use of medication among residents with hypertension.

### 3.2. Security Parameters

**Figure 3:**
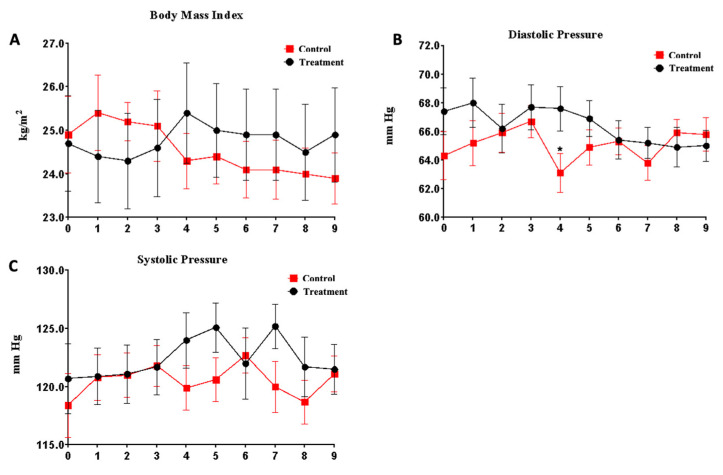
Evaluation of security parameters related to the product administration for 36 weeks. (**A**) Evaluation of body mass index. (**B**) Measurement of diastolic pressure. (**C**) Measurement of systolic pressure. * *p* < 0.05.

The product was well accepted by all the volunteers, who did not report any adverse events throughout the study. Similarly, there were no significant changes in weight (Figure 3A) and blood pressure (Figure 3B,C).

### 3.3. Respiratory Tract Infections Incidence and Related Symptoms

The incidence of respiratory infections during the intervention period was analyzed according to the appearance of symptoms related to these diseases. However, given the clinical relevance that COVID-19 presented during the period in which the study was carried out (October 2020–July 2021), it was requested that all suspected cases of this disease be confirmed by a PCR test. All the cases that appeared with symptoms compatible with COVID-19 during the 36 weeks were negative in the PCR test, so it is considered that the incidence of cases of this disease in the study was zero. Once the result of the PCR test was verified, the diseases were diagnosed: ILI and the common cold were diagnosed by the symptoms indicated in the Section 2 and the rest were classified as other viral respiratory infections (OVRI) or as bacterial respiratory infections (BRI) by medical diagnosis. Consumption of the *Alliaceae* mixture induced a significant decrease in respiratory infections, especially for ILI and the common cold, since the incidence was significantly lower than four-fold for the treatment group compared to the control group (Table 2).

The symptoms experienced by the volunteers who had RDIO episodes during the study were also analyzed. The incidence of symptoms was followed by counting the number of events suffered by the volunteers during the study (Table 3). As indicated, 14 symptoms related to ILI, the common cold, and COVID-19 were analyzed. However, one of them, the loss of smell/taste, was not observed in any of the volunteers throughout the study, and then it was removed from the statistical analysis. The intake of antibiotics or analgesics to manage respiratory infections was similar in treated and control groups during the period of intervention [IRR (95% CI): 1.449 (0.733–2.949) and 0.263 (0.009–4.179), respectively]. In addition, safety-related parameters such as nausea (1 case in each group), lack of appetite (8 cases in the control group vs. 3 in the treatment group), and sleep problems (5 cases in the control group vs. 0 in the treatment group) were lower in the treatment group (Table 3).

### 3.4. Symptom Severity

The severity of the events was analyzed by counting the number of symptoms experienced by each volunteer (Table 4) and for how many days the symptoms lasted (Table 5). In all cases, the treatment group had a lower number of symptom events than the control group, being statistically significant for cough, bone pain, fatigue, shortness of breath, and lack of appetite. In addition, it can be stated that the severity of RDIO episodes was significantly lower since the number of symptoms of the volunteers in the treatment group was 3.2 times lower (Table 4) and their duration 3.5 times lower (Table 5).

## 4. Discussion

It is well described that people over 65 years have decreased immune system functions and are, therefore, more prone to suffering from respiratory diseases of infectious origin [15]. Thus, in 2020, the influenza virus caused more than 600,000 cases in Spain; there were almost 28,000 hospitalized, 1800 Critical Care Unit admissions, and 3900 deaths, with this population group being the most affected [13]. However, it is the SARS-CoV-2 virus that causes the disease COVID-19, which has been most relevant in recent years, mainly affecting the elderly population and especially nursing homes.

The present study was carried out during the months of October 2020 to July 2021, which included from the 2nd to the 5th wave of COVID-19 [16]. For this reason, the measures taken at the “Residencia de Mayores Claret de Granada” (Spain) were extreme: mandatory use of masks, isolation of residents, closure of common dining rooms, and prohibition of the entry of people outside the residence, among others. Moreover, all residents were vaccinated against flu and pneumonia and received two doses of the Pfizer vaccine for COVID-19. That is why the incidence of RDIO in the present study was not very high. In fact, no suspicion of COVID-19 was confirmed by a PCR test during the intervention period.

In the present study, throughout the 36 weeks of intervention, no mild or severe adverse events related to the consumption of the product were reported. In addition, the weight and blood pressure of the volunteers were followed every four weeks, and no significant changes were detected (Figure 3). In fact, the safety of similar extracts [17,18] and compounds present in *Alliaceae* [19,20,21] has been previously analyzed in several studies, obtaining similar conclusions. In relation to weight, opposite effects were seen during the study. While in the control group, BMI decreased by 5.9% (25.4 + 4.9 vs. 23.9 + 3.3 *p* = 0.875), in the treatment group, there was a slight increase of 2.05% (24.4 + 6.1 vs. 24.9 + 6.2 *p* = 0.676). This weight stabilization can be considered very positive data regarding the safety of the extract intake. In fact, weight loss is a natural process in older people. Thus, in a similar study with elderberry and reishi extracts [22] previously carried out in the same nursing home, weight decreased in both groups after 14 weeks of intervention.

There are few studies carried out in humans that report the benefits of *Allium* extracts in the prevention of infectious processes. Moreover, most of them focus on the benefits of garlic or onion separately, not studying the effects of their combination. In addition, the trials found in the literature focus on the effects of their consumption no later than 12 weeks.

A metabolomic study carried out with a similar *Allium* extract containing 14.5% of organosulfur compounds that was administered for 4 weeks to 30 healthy volunteers showed changes mainly in the phospholipid metabolism, highlighting the increase in the concentration of 7 lysoPCs, which have demonstrated a relationship with the prevention of several diseases [23]. Other garlic and onion extracts have been previously tested in humans in an uncontrolled metabolomics study targeting tumor markers and metabolic syndrome [24,25], but their protective effect against infectious respiratory diseases has never been described in human studies. In fact, only two double-blind, placebo-controlled trials have been reported. A study performed with 146 participants that consumed an allicin-containing supplement for 12 weeks revealed that its intake can reduce the incidence and prevent the associated symptoms of the common cold [26]. In line with these results, another similar study that included 65 patients diagnosed with COVID-19 showed that the intake of allicin capsules for 2 weeks could have a significant impact on improving clinical symptoms and accelerating the healing process [27]. Thus, a higher potential activity of garlic extract rich in allicin in controlling viral infections has been reported [28].

It is important to highlight that different compounds from either garlic or onion can act synergically to exert beneficial effects after their administration. Thus, allicin from garlic is extremely unstable and is rapidly transformed in other compounds such as dithiins or diallyl disulfide [29], but other organosulfur compounds found in garlic, like alliin, can contribute to its beneficial effects due to the reported anti-inflammatory properties [30]. In onion, the most common sulfur compound is propiin (S-propyl-L-cysteine sulfoxide) that, due to the action of alliinase, leads to propiin derivatives, such as thiosulfinates and thiosulfonates compounds such as PTS and PTSO, which are more stable and exhibit interesting biological properties as antimicrobial, anti-inflammatory, or antitumor activities [31,32,33]. Although there is no evidence in the literature on its ability to prevent infectious processes in humans, there are many preclinical studies that support our results. Accordingly, different studies with these propiin derivatives have been carried out in farm animals, which have demonstrated protective effects against infections in broiler chickens [34], fish [10], and pigs [35,36]. Furthermore, these compounds have demonstrated the ability to modulate the gut microbiome in aquatic and terrestrial animals, promoting the development of lactic acid bacteria to the detriment of enteropathogenic bacteria [10,30,37], improving host immunity, maintaining intestinal homeostasis, and inhibiting inflammation [38].

While most of the studies based on the intake of garlic linked their effects to the action of allicin, in the case of onion, the studies related their benefits to quercetin [39]. The antiviral activity of this compound against influenza viruses, rhinovirus, and SARS-CoV-2 virus has also been studied [40,41,42]. In contrast, only a few studies have been carried out on the benefits of the organosulfur compounds from onion.

As previously mentioned, respiratory infections caused by bacteria, rhinovirus, coronavirus, and influenza virus, among others, affect the respiratory and immune systems; common in these diseases is a high inflammation that affects almost all patients [43]. Onion is a food well known for its anti-inflammatory properties [44]. Some works have recently reported the anti-inflammatory effect of thiosulfinate and thiosulfonate from onion in human cells [33]. Besides, these compounds derived from propiin demonstrated in preclinical trials their ability to modulate the inflammatory response and their potential applications in bowel auto-immune diseases and intestinal infections [9,45]. Therefore, onion can also play an essential role in boosting the immune system, improving lung function, and could prevent respiratory infections [45].

According to this approach, some researchers have recently hypothesized that onion consumption could be a good candidate for managing COVID-19 patients due to its anti-inflammatory, antithrombotic, and antiviral effect [46,47]. Other authors have also informed on the therapeutic potential of onion against acute respiratory tract infection and lung injury caused by collagen deposition, inflammatory cell infiltration, and pulmonary fibrosis [48].

Therefore, this study is the first controlled, randomized, and double-blind study conducted with a mixture of garlic and onion extract concentrate, demonstrating its protective effect on respiratory diseases of infectious origin in elderly healthy volunteers in residential care facilities, evidenced by a decrease in the number of events of infectious respiratory diseases, as well as in the number of associated symptoms and their duration. In conclusion, regular consumption of an *Allium* extract improves the immunity of elderly volunteers and can be used prophylactically against the most common infectious respiratory diseases.

## 5. Limitations and Strengths

The main limitation of this study is related to the fact that this was not a multicentric study since the possibility of including additional participants from other nursing homes was limited due to the pandemic during the study period.

Moreover, the population distribution in the elderly centers constitutes another limitation since more females participated in the study. Although these limitations could have had an impact on our findings, it is unlikely that they could have altered the general conclusions. Another limitation is related to the severity of the symptoms. These were not evaluated because SARS-CoV-2 preventive measures would not allow a real evaluation of the severity of the symptoms. In addition, the Ethical Committee did not approve the acquisition of blood samples; therefore, the measurement of biochemical parameters that could have explained the effects and safety of the treatment could not be carried out. As this point was very important, safety was indirectly monitored by analyzing various parameters, such as nausea, appetite, sleep, blood pressure, weight, and consumption of other medications.

Lastly, most of the subjects included in this trial were smokers and drinkers, and although these habits were more common than in the general population, the distribution of smokers and drinkers in both study groups was similar, and consequently, it can be assumed that these habits did not influence the results.

The manuscript also has an important strength, as it was conducted with institutionalized elderly people, which allowed for a highly controlled environment in all aspects: habits, diet, and drug intake therapy, including the product of the study itself, allowing for immediate control of any adverse effects.

## Figures and Tables

**Table 1:** Descriptive values of the study volunteers. Continuous variables are presented as the mean ± SD and categorical variables as *n* (%). *p*-value indicates statistical differences between groups.

	Control Group (*n* = 33)	Treatment Group (*n* = 32)	*p*-Value
Age (years)	84.5 ± 8.0	84.3 ± 10.1	0.940
Gender			0.537
Men	8 (24.2)	5 (15.6)	
Women	25 (75.8)	27 (84.4)	
Body Mass Index BMI (kg/m^2^)	24.9 ± 5.0	24.7 ± 6.3	0.881
Smoking habits			0.873
Current smoker	29 (87.9)	27 (84.4)	
Former smoker	2 (6.1)	3 (9.4)	
No smoker	2 (6.1)	2 (6.2)	
Alcoholic drinks			1.000
Regular drinker	30 (90.9)	30 (93.8)	
Former drinker	2 (6.1)	1 (3.1)	
Non drinker	1 (3.0)	1 (3.1)	
Physical activity			0.762
Very low	15 (45.4)	13 (40.6)	
Low	18 (54.6)	19 (59.4)	
Systolic pressure (mmHg)	118.4 ± 15.5	120.7 ± 17.4	0.571
Diastolic pressure (mmHg)	64.3 ± 9.5	67.4 ± 9.4	0.191

**Table 2:** Respiratory infection cases. ORVI: other respiratory viral infections. RBI: respiratory bacterial infections. Ratio ns/s: ratio no symptoms vs. symptoms. IRR c/t: incidence rate/ratio control group vs. treatment group. NC: no cases (IRR not possible).

	Group	Number of Events				Statistical Analysis			
		0	1	2	3	Ratio ns/s	*p*-Value	IRR c/t	*p*-Value
Influenza	Control	21	9	3	0	0.013	0.005	3.717	0.021
	Treatment	29	1	2	0	0.004	0.002		
Common cold	Control	18	8	2	5	0.021	0.007	4.038	0.001
	Treatment	28	2	0	2	0.005	0.003		
ORVI	Control	29	4	0	0	0.004	0.002	2.468	0.352
	Treatment	30	2	0	0	0.002	0.001		
RBI	Control	25	7	1	0	0.006	0.003	1.943	0.263
	Treatment	29	2	0	1	0.003	0.002		

**Table 3:** Total number of incidents throughout the study. Ratio ns/s: ratio no symptoms vs. symptoms. IRR c/t: incidence rate/ratio control group vs. treatment group. NC: no cases (IRR not possible).

Symptoms	Group	Number of Events				Statistical Analysis			
		0	1	2	3	Ratio ns/s	*p*-Value	IRR c/t	*p*-Value
Cough	Control	18	12	3	0	0.014	0.005	4.792	0.005
	Treatment	29	2	0	1	0.003	0.002		
Fever	Control	27	5	1	0	0.009	0.005	6.450	0.093
	Treatment	31	1	0	0	0.001	0.001		
Nasal congestion	Control	27	6	0	0	NC	NC	NC	NC
	Treatment	32	0	0	0	NC	NC		
Throat pain	Control	30	3	0	0	0.001	0.001	1.719	0.599
	Treatment	30	1	1	0	0.001	0.001		
Headache	Control	29	4	0	0	NC	NC	NC	NC
	Treatment	32	0	0	0	NC	NC		
Bone pain	Control	28	4	1	0	0.002	0.002	12.465	0.033
	Treatment	31	0	1	0	0.000	0.000		
Fatigue	Control	22	6	3	2	0.020	0.007	5.235	0.004
	Treatment	29	1	2	0	0.004	0.002		
Chest pain	Control	27	4	2	0	0.006	0.003	2.641	0.185
	Treatment	28	2	1	0	0.002	0.002		
Difficulty breathing	Control	20	8	4	1	0.016	0.006	3.751	0.007
	Treatment	28	2	1	1	0.004	0.002		
Nausea	Control	32	1	0	0	0.000	0.000	1.407	0.818
	Treatment	31	1	0	0	0.000	0.000		
Diarrhea	Control	27	4	1	1	0.007	0.004	1.674	0.389
	Treatment	28	3	1	0	0.004	0.003		
Lack of appetite	Control	25	5	3	0	0.014	0.006	3.720	0.058
	Treatment	29	3	0	0	0.004	0.003		
Sleeping problems	Control	28	5	0	0	NC	NC	NC	NC
	Treatment	32	0	0	0	NC	NC		

**Table 4:** Number of symptoms experienced by the volunteers. Ratio ns/s: ratio no symptoms vs. symptoms. IRR c/t: Incidence rate/ratio control group vs. treatment group.

Group	Number of Symptoms															
	0	1	2		3	4	5	6	7	8		9	11	12	13	17
Control	13	5	3		0	1	3	0	1	2		1	1	1	1	1
Treatment	23	4	0		2	0	0	1	0	1		0	0	1	0	0
				Statistical analysis												
				Ratio ns/s			*p*-value		IRR c/t		*p*-value					
		Control		0.110			0.010		3.231		0.000					
		Treatment		0.034			0.006									

**Table 5:** Total number of days with symptoms. Ratio ns/s: ratio no symptoms vs. symptoms. IRR c/t: incidence rate/ratio control group vs. treatment group.

Group	Days																		
	0	1	2	3	4	6	7		8	9	10	15	17		18	21	22	24	56
Control	13	1	3	1	3	2	1		0	1	3	1	1		1	0	0	1	1
Treatment	23	2	1	1	0	0	0		1	0	2	0	0		0	1	1	0	0
					Statistical analysis														
					Ratio ns/s			*p*-value		IRR c/t				*p*-value					
		Control			0.168			0.019		3.508				0.000					
		Treatment			0.048			0.008											

## Data Availability

The research data for this study have been obtained by Carlos Gracián from the electronic file that Residencia Claret de Granada has. Since it is a confidential file, any questions about the study data should be made to him.

## References

[1] United Nations (2019). World Population Ageing 2019 Highlights.

[2] Crighton E.J., Elliott S.J., Moineddin R., Kanaroglou P., Upshur R.E. (2007). An exploratory spatial analysis of pneumonia and influenza hospitalizations in Ontario by age and gender. Epidemiol. Infect..

[3] Falsey A.R., Walsh E.E. (2006). Viral pneumonia in older adults. Clin. Infect. Dis..

[4] McElhaney J.E., Zhou X., Talbot H.K., Soethout E., Bleackley R.C., Granville D.J., Pawelec G. (2012). The unmet need in the elderly: How immunosenescence, CMV infection, comorbidities and frailty are a challenge for the development of more effective influenza vaccines. Vaccine.

[5] Loeb M.B. (2005). Pneumonia in nursing homes and long-term care facilities. Semin. Respir. Crit. Care Med..

[6] Anne S., Gerhard P. (2020). Health, safety and quality concerns of plant-based traditional medicines and herbal remedies. S. Afr. J. Bot..

[7] Hafiz Ansar Rasul S., Masood Sadiq B., Nauman K., Saira S., Ali R., Muhammad A., Munawar A. (2015). Garlic (*Allium sativum*): Diet based therapy of 21st century—A review. Asian Pac. J. Trop. Dis..

[8] Vezza T., Garrido-Mesa J., Díez-Echave P., Hidalgo-García L., Ruiz-Malagón A.J., García F., Sánchez M., Toral M., Romero M., Duarte J. (2021). *Allium*-Derived Compound Propyl Propane Thiosulfonate (PTSO) Attenuates Metabolic Alterations in Mice Fed a High-Fat Diet through Its Anti-Inflammatory and Prebiotic Properties. Nutrients.

[9] Vezza T., Algieri F., Garrido-Mesa J., Utrilla M.P., Rodríguez-Cabezas M.E., Baños A., Guillamón E., García F., Rodríguez-Nogales A., Gálvez J. (2019). The Immunomodulatory Properties of Propyl-Propane Thiosulfonate Contribute to its Intestinal Anti-Inflammatory Effect in Experimental Colitis. Mol. Nutr. Food Res..

[10] Cabello-Gómez J.F., Aguinaga-Casanas M.A., Falcón-Piñeiro A., González-Gragera E., Márquez-Martín R., Agraso M.D.M., Bermúdez L., Baños A., Martínez-Bueno M. (2022). Antibacterial and Antiparasitic Activity of Propyl-Propane-Thiosulfinate (PTS) and Propyl-Propane-Thiosulfonate (PTSO) from *Allium cepa* against Gilthead Sea Bream Pathogens in In Vitro and In Vivo Studies. Molecules.

[11] Magrys A., Olender A., Tchorzewska D. (2021). Antibacterial properties of *Allium sativum* L. against the most emerging multidrug-resistant bacteria and its synergy with antibiotics. Arch. Microbiol..

[12] Liu Y., Che T.M., Song M., Lee J.J., Almeida J.A., Bravo D., Van Alstine W.G., Pettigrew J.E. (2013). Dietary plant extracts improve immune responses and growth efficiency of pigs experimentally infected with porcine reproductive and respiratory syndrome virus. J. Anim. Sci..

[13] European Centre for Disease Prevention and Control. https://www.ecdc.europa.eu/en/all-topics/eu-case-definitions.

[14] Eccles R. (2005). Understanding the symptoms of the common cold and influenza. Lancet Infect. Dis..

[15] Wu Y., Goplen N.P., Sun J. (2021). Aging and respiratory viral infection: From acute morbidity to chronic sequelae. Cell. Biosci..

[16] Evolucíon Pandemia. https://cnecovid.isciii.es/covid19/#evoluci%C3%B3n-pandemia.

[17] Mellado-García P., Puerto M., Prieto A.I., Pichardo S., Martín-Cameán A., Moyano R., Blanco A., Cameán A.M. (2016). Genotoxicity of a thiosulfonate compound derived from *Allium* sp. intended to be used in active food packaging: In vivo comet assay and micronucleus test. Mutat. Res. Genet. Toxicol. Env. Mutagen..

[18] Mellado-García P., Maisanaba S., Puerto M., Prieto A.I., Marcos R., Pichardo S., Cameán A.M. (2017). In vitro toxicological assessment of an organosulfur compound from *Allium* extract: Cytotoxicity, mutagenicity and genotoxicity studies. Food Chem. Toxicol..

[19] Llana-Ruiz-Cabello M., Maisanaba S., Gutiérrez-Praena D., Prieto A.I., Pichardo S., Jos A., Moreno F.J., Cameán A.M. (2015). Cytotoxic and mutagenic in vitro assessment of two organosulfur compounds derived from onion to be used in the food industry. Food Chem..

[20] Lira A.C., Prieto A.I., Baño S.A., Guillamón E., Moyano, Jos A., Cameán A.M. (2020). Safety assessment of propyl-propane-thiosulfonate (PTSO): 90-days oral subchronic toxicity study in rats. Food Chem. Toxicol..

[21] Cascajosa-Lira A., Pichardo S., Baños A., Guillamón E., Molina-Hernández V., Moyano R., Jos Á., Cameán A.M. (2022). Acute and subchronic 90-days toxicity assessment of propyl-propane-thiosulfinate (PTS) in rats. Food Chem. Toxicol..

[22] Gracián-Alcaide C., Maldonado-Lobón J.A., Ortiz-Tikkakoski E., Gómez-Vílchez A., Fonollá J., López-Larramendi J.L., Olivares M., Blanco-Rojo R. (2020). Effects of a Combination of Elderberry and Reishi Extracts on the Duration and Severity of Respiratory Tract Infections in Elderly Subjects: A Randomized Controlled Trial. Appl. Sci..

[23] Fernández-Ochoa A., Borrás-Linares I., Baños A., García-López J.D., Guillamón E., Nuñez-Lechado C., Quirantes-Piné R., Segura A. (2018). A fingerprinting metabolomic approach reveals deregulation of endogenous metabolites after the intake of a bioactive garlic supplement. J. Funct. Foods.

[24] Ishikawa H., Saeki T., Otani T., Suzuki T., Shimozuma K., Nishino H., Fukuda S., Morimoto K. (2006). Aged garlic extract prevents a decline of NK cell number and activity in patients with advanced cancer. J. Nutr..

[25] Matsumoto S., Nakanishi R., Li D., Alani A., Rezaeian P., Prabhu S., Abraham J., Fahmy M.A., Dailing C., Flores F. (2016). Aged Garlic Extract Reduces Low Attenuation Plaque in Coronary Arteries of Patients with Metabolic Syndrome in a Prospective Randomized Double-Blind Study. J. Nutr..

[26] Josling P. (2001). Preventing the common cold with a garlic supplement: A double-blind, placebo-controlled survey. Adv. Ther..

[27] Yaghoubian H., Niktale H., Yazdi A.P., Ghorani V., Rashed M.M., Hashemian A.M. (2021). Evaluate the Therapeutic Effect of Allicin (L-cysteine) on Clinical Presentation and Prognosis in Patients with COVID-19. Eur. J. Transl. Myol..

[28] Rouf R., Uddin S.J., Sarker D.K., Islam M.T., Ali E.S., Shilpi J.A., Nahar L., Tiralongo E., Sarker S.D. (2020). Antiviral potential of garlic (*Allium sativum*) and its organosulfur compounds: A systematic update of pre-clinical and clinical data. Trends Food Sci. Technol..

[29] Guillamón E., Andreo-Martínez P., Mut-Salud N., Fonollá J., Baños A. (2021). Beneficial Effects of Organosulfur Compounds from Allium cepa on Gut Health: A Systematic Review. Foods.

[30] Sánchez-Sánchez M.A., Zepeda-Morales A.S.M., Carrera-Quintanar L., Viveros-Paredes J.M., Franco-Arroyo N.N., Godínez-Rubí M., Ortuño-Sahagun D., López-Roa R.I. (2020). Alliin, An Allium sativum Nutraceutical, Reduces Metaflammation Markers in DIO Mice. Nutrients.

[31] Keusgen M., Schulz H., Glodek J., Krest I., Krüger H., Herchert N., Keller J. (2002). Characterization of SomeAlliumHybrids by Aroma Precursors, Aroma Profiles, and Alliinase Activity. J. Agric. Food Chem..

[32] Sorlozano-Puerto A., Albertuz-Crespo M., Lopez-Machado I., Ariza-Romero J.J., Baños-Arjona A., Exposito-Ruiz M., Gutierrez-Fernandez J. (2018). In Vitro Antibacterial Activity of Propyl-Propane-Thiosulfinate and Propyl-Propane-Thiosulfonate Derived from Allium spp. against Gram-Negative and Gram-Positive Multidrug-Resistant Bacteria Isolated from Human Samples. Biomed. Res. Int..

[33] Guillamón E., Mut-Salud N., Rodríguez-Sojo M.J., Ruiz-Malagón A.J., Cuberos-Escobar A., Martínez-Férez A., Rodríguez-Nogales A., Gálvez J., Baños A. (2023). In Vitro Antitumor and Anti-Inflammatory Activities of Allium-Derived Compounds Propyl Propane Thiosulfonate (PTSO) and Propyl Propane Thiosulfinate (PTS). Nutrients.

[34] Kim D.K., Lillehoj H.S., Lee S.H., Jang S.I., Lillehoj E.P., Bravo D. (2013). Dietary Curcuma longa enhances resistance against Eimeria maxima and *Eimeria tenella* infections in chickens. Poult. Sci..

[35] Ruiz R., García M.P., Lara A., Rubio L.A. (2010). Garlic derivatives (PTS and PTS-O) differently affect the ecology of swine faecal microbiota in vitro. Vet. Microbiol..

[36] Liu Y. (2015). Fatty acids, inflammation and intestinal health in pigs. J. Anim. Sci. Biotechnol..

[37] Rabelo-Ruiz M., Ariza-Romero J.J., Zurita-González M.J., Martín-Platero A.M., Baños A., Maqueda M., Valdivia E., Martínez-Bueno M., Peralta-Sánchez J.M. (2021). Allium-Based Phytobiotic Enhances Egg Production in Laying Hens through Microbial Composition Changes in Ileum and Cecum. Animals.

[38] Shi N., Li N., Duan X., Niu H. (2017). Interaction between the gut microbiome and mucosal immune system. Mil. Med. Res..

[39] Maurya P.K. (2022). Health Benefits of Quercetin in Age-Related Diseases. Molecules.

[40] Naithani R., Huma L.C., Holland L.E., Shukla D., McCormick D.L., Mehta R.G., Moriarty R.M. (2008). Antiviral activity of phytochemicals: A comprehensive review. Mini Rev. Med. Chem..

[41] Ganesan S., Faris A.N., Comstock A.T., Wang Q., Nanua S., Hershenson M.B., Sajjan U.S. (2012). Quercetin inhibits rhinovirus replication in vitro and in vivo. Antivir. Res..

[42] Gasmi A., Mujawdiya P.K., Lysiuk R., Shanaida M., Peana M., Gasmi Benahmed A., Beley N., Kovalska N., Bjørklund G. (2022). Quercetin in the Prevention and Treatment of Coronavirus Infections: A Focus on SARS-CoV-2. Pharmaceutics.

[43] Vahid F., Rahmani D. (2021). Can an anti-inflammatory diet be effective in preventing or treating viral respiratory diseases? A systematic narrative review. Clin. Nutr. ESPEN.

[44] Marefati N., Eftekhar N., Kaveh M., Boskabadi J., Beheshti F., Boskabady M.H. (2018). The Effect of Allium cepa Extract on Lung Oxidant, Antioxidant, and Immunological Biomarkers in Ovalbumin-Sensitized Rats. Med. Princ. Pract..

[45] Zhu L., Myhill L.J., Andersen-Civil A.I.S., Thamsborg S.M., Blanchard A., Williams A.R. (2022). Garlic-Derived Organosulfur Compounds Regulate Metabolic and Immune Pathways in Macrophages and Attenuate Intestinal Inflammation in Mice. Mol. Nutr. Food Res..

[46] Dorsch W., Ring J. (2020). Anti-inflammatory substances from onions could be an option for treatment of COVID-19—A hypothesis. Allergo J..

[47] Haslberger A.G., Jakob U., Hippe B., Karlic H. (2020). Mechanisms of selected functional foods against viral infections with a view on COVID-19, Mini review. Funct. Foods Health Dis..

[48] Thota S.M., Balan V., Sivaramakrishnan V. (2020). Natural products as home-based prophylactic and symptom management agents in the setting of COVID-19. Phytother. Res..

